# Changes in resting-state functional connectivity in patients with congenital adrenal hyperplasia

**DOI:** 10.1016/j.nicl.2022.103081

**Published:** 2022-06-09

**Authors:** Valeria Messina, Annelies van't Westeinde, Nelly Padilla, Svetlana Lajic

**Affiliations:** a Department of Women’s and Children’s Health, Karolinska Institutet, Pediatric Endocrinology Unit (QB83), Karolinska University Hospital, SE-171 76 Stockholm, Sweden; b Department of Women’s and Children’s Health, Karolinska Institutet, Tomtebodavägen 18b, SE- 171 77 Stockholm, Sweden; c Karolinska University Hospital, SE- 171 76 Stockholm, Sweden

**Keywords:** **CAH**, congenital adrenal hyperplasia, **GC**, glucocorticoid, **MC**, mineralocorticoid, **SW**, salt-wasting, **SV**, simple virilising, **NC**, non-classic, **WAIS**, Wechsler Adult Intelligence Scale, **WISC**, Wechsler Intelligence Scales for Children, **WMS**, Wechsler Memory Scale, **RSNs**, resting-state networks, **rs-fMRI**, resting-state functional magnetic resonance imaging, **DMN**, default-mode network, **PCC**, posterior cingulate cortex, **PFC**, prefrontal-cortex, **ICA**, independent component analyses, CAH, Resting state, Brain function, Cortisol, Default mode network

## Abstract

**Context:**

Patients with congenital adrenal hyperplasia (CAH) are treated with life-long glucocorticoid (GC) replacement therapy. Negative effects on cognition, brain structure and function during working memory tasks have been identified. To date, no studies on functional connectivity during rest have been performed in patients with CAH.

**Objective:**

To investigate resting-state functional connectivity in patients with CAH compared with healthy untreated controls and the association between functional connectivity in the precuneus and disease severity, dose of GC and working memory (WM).

**Design, Setting and Participants:**

Thirty-one patients with CAH (18 females) and 38 healthy controls (24 females), aged 16–33 years, from a single research institute, underwent functional magnetic resonance imaging of the brain during rest.

**Results:**

Patients with CAH showed increased functional connectivity in the precuneus compared with controls. Post-hoc tests within the precuneus showed that only patients with simple virilising CAH had stronger connectivity compared to controls. Further, while both patients with salt-wasting and simple virilising CAH performed worse on a WM task compared to controls, functional connectivity in the precuneus was not associated with executive function performance.

**Conclusion:**

Patients with CAH demonstrated altered functional connectivity during rest in the precuneus. Such a change may reflect a functional reorganisation in response to the CAH disease. The change in functional connectivity may depend on the severity of CAH.

## Highlights

• Patients with CAH showed increased functional connectivity during rest in the precuneus compared with controls.
• This change may reflect a functional reorganisation in response to the CAH disease.
• The change in functional connectivity may also depend on the severity of CAH.

## 1 Introduction

Patients with classic congenital adrenal hyperplasia (CAH) due to 21-hydroxylase deficiency, a disorder characterised by impaired cortisol and mineralocorticoid (MC) synthesis in the adrenal gland, require adequate treatment with glucocorticoids (GCs) and MCs to avoid adrenal/salt-losing crises. Despite close monitoring, it is difficult to reach an exact GC dosing to regulate the hypothalamic–pituitaryadrenal (HPA) axis adequately and reflect the circadian and ultradian rhythm of cortisol secretion (Speiser et al., 2018). Thus, patients with CAH may experience episodes of over- or under-treatment, with a risk of adverse effects on metabolism (Volkl et al., 2006, Falhammar et al., 2007, Falhammar et al., 2011), cognition (Johannsen et al., 2006, Browne et al., 2015, Karlsson et al., 2017); behaviour (Pasterski et al., 2007, Frisen et al., 2009, Engberg et al., 2015) and brain structure and function (Van't Westeinde et al., 2020, Mazzone et al., 2011).

Most studies of patients with CAH have focused on the long-term effects of GC exposure on cognitive domains, suggesting deficits in verbal and visuo-spatial working memory, lower intelligence and an increased risk of psychiatric disorders, especially in patients with the most severe salt-wasting (SW) phenotype (Idris et al., 2014, Falhammar et al., 2014, Johannsen et al., 2006, Browne et al., 2015, Karlsson et al., 2017).

Cortisol acts through the GC and MC receptors expressed throughout the brain, particularly in key regions important for memory functions, emotional regulation and executive functions, such as the hippocampus, amygdala and prefrontal cortex that are vulnerable to high doses of GC (Funahashi, 2001, LeDoux, 2000, Opitz, 2014). Cortisol, crucial for the regulation of glucose metabolism, can also affect excitatory glutamate signaling, especially in brain areas rich in glutamate receptors (e.g., the prefrontal cortex) (Yuen et al., 2009). Therefore, long-term disturbances in cortisol levels may impact widespread brain networks, especially highly connected areas (i.e. structural hubs).

Results from several studies have indicated that prolonged exposure to high GC levels, either by stress or synthetic GCs, may cause impairment in brain function or brain structure (Fuchs and Flugge, 1998, Van't Westeinde et al., 2019, Stomby et al., 2019). However, studies of brain structure and function in patients with CAH compared with controls remain scarce.

Webb and colleagues reported changes in white matter microstructure and volume of the right hippocampus, cerebellum and brainstem. The authors also reported an association between current GC replacement therapy and cognitive abnormalities in women with CAH, suggesting that the GC replacement regimen has a profound impact on brain morphology and function (Webb et al., 20182018).

We recently showed in our systematic study on brain morphology in patients with CAH (aged 16–33 years) that CAH is associated with brain structural alterations in both males and females. Of note, we observed reduced cortical thickness in grey matter in the middle frontal gyrus and the parietal and superior occipital cortex, key regions of the working memory networks. We also found a reduced volume of the precuneus (Van't Westeinde et al., 2019). Both the middle frontal gyrus and the precuneus play an important role in executive functions. The precuneus is part of both the default mode network (DMN) and the central executive network, and is involved in switching attention between those networks, as well as higher cognitive processes, such as episodic memory retrieval, decision making, mental rotation and exemplar-based judgment processes (Cavanna and Trimble, 2006, Kaboodvand et al., 2018, Riemer et al., 2020, Stillesjo et al., 2019). The structural alterations that we found in our study on brain morphology may affect the organisation of the brain and therefore may reflect changes in the functional connectivity of brain networks in individuals with CAH.

Resting-state functional magnetic resonance imaging (rs-fMRI) has been used to investigate the activity of interconnected brain regions or networks during rest, namely resting-state networks (RSNs) (Doucet et al., 2011, Smith et al., 2009). The major RSNs described in the literature in healthy individuals include visual, audition/language, sensorimotor, attention, salience, DMN and the central executive networks (Smith et al., 2009, Petersen and Sporns, 2015). Each network has its function and continuously shares information with the others, playing an essential role in a wide range of cognitive functions (Smith et al., 2009). The main networks involved in cognitive functions are the DMN, central executive and salience network (triple network model). Abnormalities in one core network may impact the other two (Menon, 2011).

The DMN, active during the resting state, is the largest brain network (Raichle, 2015, Greicius et al., 2003). Recent studies have shown that the DMN cannot be defined as a single network but comprises areas working in concert and distributed across the brain, each specialised in a specific processing domain (Raichle, 2015, Buckner and DiNicola, 2019). These regions of the DMN, including large parts of the prefrontal cortex, posteromedial and inferior parietal cortex and parts of the temporal cortex, are functionally correlated (Greicius et al., 2003), showing synchronous activity patterns. The DMN is active during passive sensory tasks with low cognitive demand, i.e. the mind is continuously wandering stereotypically. Conversely, the DMN is deactivated during a cognitive task that requires external attention. More particularly, it has been observed that the posterior cingulate cortex (PCC), which plays a central role in the DMN and is characterised by high energy demand during rest, shows decreased activity during a working memory task (Buckner and DiNicola, 2019). Moreover, the regions of the DMN are negatively correlated with regions involved in attention (Fox and Raichle, 2007), which are part of the “dorsal attention system” (Corbetta and Shulman, 2002).

As opposed to the DMN, the central executive network, whose nodes include the dorsolateral prefrontal cortex and the posterior parietal cortex, is activated during a cognitive task requiring attention. Of note, these two regions are anticorrelated, implying that they are activated and deactivated together, suggesting that they are involved in different cognitive tasks (Menon, 2011).

The salience network, comprising the anterior insula and the dorsal anterior cingulate cortex, is responsible for switching between the DMN and central executive network, as it has been shown from the triple network model (Menon, 2011). It plays a key function when identifying internal and external stimuli to guide behaviour (Chand et al., 2017).

In a recent study investigating differences in functional activation of the brain during a verbal and visuo-spatial working memory task in patients with CAH we found increased activity in occipital and parietal regions (left angular gyri and precuneus) of the working memory network in males (Van't Westeinde, 2021, submitted), suggesting that long-term GC imbalances may affect brain functions.

Few studies have investigated the effects of long-term cortisol disturbances on functional connectivity during rest. One study on resting-state functional connectivity conducted in patients with Cushing’s syndrome suggests that long-term cortisol excess results in increased connectivity in the DMN, medial temporal lobe and prefrontal cortex (Stomby et al., 2019). To our knowledge, no studies on patients with CAH have been performed or on other patient groups treated with exogenous GC.

Thus, in this exploratory study we aim to investigate the whole-brain resting-state functional connectivity in patients with CAH compared with controls from the general population using independent component analysis (ICA). Based on our previous findings of differences in brain structures in both the DMN and working memory network, we expected to find altered functional connectivity during rest in patients with CAH, especially in the DMN. In addition, we tested the association between functional connectivity and executive functions.

Moreover, we also conduct post-hoc tests to investigate the association between functional connectivity in our region of interest (ROI), the precuneus, and disease severity (phenotype), GC dose at the time of testing and the performance in a visuo-spatial working-memory test was investigated.

## 2 Material and Methods

### 2.1 Participants

The recruitment process has been described elsewhere (Karlsson et al., 2017, Van't Westeinde et al., 2020). All the participants (adolescents and adults) of this report were part of a larger longitudinal study investigating short- and long-term effects of pre- and postnatal treatment with GC in patients with CAH due to 21-hydroxylase deficiency (CYP21A2) (the PREDEX study). The entire PREDEX cohort comprises children and adults with CAH, participants at risk of CAH treated prenatally with dexamethasone, both with and without CAH, and healthy untreated controls from the general population. For the present article, we focused on patients with CAH that did not receive prenatal dexamethasone treatment and healthy, untreated controls aged 16–33 years (y).

Among the CAH patients invited to the MRI study, 61.1% agreed to participate; 26.3% of the contacted population controls participated in the study. After participation, patients with a reported history of neuropsychological problems and/or current medication for a psychiatric disorder or central stimulant treatment were not included in the analyses (two CAH patients, five controls). Moreover, 8 participants (three CAH patients, five controls) were excluded from the analyses because of excessive motion artifacts during the scanning sessions, as explained in the image analysis section. One participant was excluded because of enlarged ventricles and another because of signal loss in the frontal cortex related to metallic braces. Thus, the final sample consisted of 31 patients with CAH (18 females) and 38 controls (24 females). Two patients in the CAH group had non-classic (NC) CAH, 13 had simple-virilising (SV) CAH and 16 had SW CAH. All patients were treated with GC and 25/31 patients had MC replacement therapy. The metabolic control of the patients was monitored in 23/31 patients on a regular basis during clinical check-ups with 24-h blood profiles of 17-hydroxyprogesterone (sampling before each dose of GC). For 83% of the patients the metabolic control was adequate. In the CAH group there were two pairs of siblings. In the control group two participants were siblings to two individuals in the CAH group. The age range of all the participants was 16–33 y (mean age = 21.9 y, standard deviation = 4.1 y). Groups differed in age, with CAH patients being approximately 3 years older than controls (mean age, CAH = 23.8 y, standard deviation = 4.8 y, range = 16–33 y; mean age, controls = 20.5 y, standard deviation = 2.7 y, age range = 16–25 y). Demographic data and clinical background data are presented in Table 1. All participants, and parents of minors, were initially contacted by an invitation letter and gave their informed written consent. The study protocol, recruitment and scanning procedures were approved by the Regional Ethics Committee of Karolinska Institutet and Stockholm (Dnr 99–153, and 2011/1764–32).

**Table 1:** **Demographic and clinical data for individuals with CAH and population controls (C).** Data are shown as mean (±SD). F-statistics and *p*-values are given to compare CAH *vs*. controls.

	Female groups		Male groups		CAH	
	CAH [f]	C [f]	CAH [m]	C [m]	F statistics	*p values*
N	18	24	13	14		
Age	24.3 (4.5)	20.2 (2.4)	23.1 (5.5)	21.0 (3.0)	12.7	**0.001**
Subject Education 1	2.1 (0.7)	1.7 (0.6)	2.2 (0.6)	1.9 (0.5)	0.26	0.821
Well-being 2	7.3 (2.0)	7.5 (0.9)	7.4 (1.6)	7.3 (2.0)	0.26	0.264
HC (mg/m^2^)	12.9 (4.2)		15.2 (4.8)			
Fludrocortisone (μg/day)	122.7 (51.8)		112.5 (43.3)			
Heigth (cm)	162.1 (6.2)	169.2 (6.6)	172.8 (7.3)	180.2 (4.5)	10.58	**0.002**
Weight (kg)	67.2 (12.7)	65.1 (10.4)	75.8 (13.8)	80.8 (14.8)	0.02	0.880
BMI	25.7 (5.4)	22.7 (3.1)	25.4 (4.6)	24.5 (3.9)		**0.044**
Nonparametric tests					***U test***^6^ *Total group*	
Alcohol 3	1.1 (0.9)	0.9 (0.9)	1.1 (0.9)	1.0 (1.0)	0.516	
Drugs 4	0.0 (0.0)	0.0 (0.0)	0.0 (0.0)	0.1 (0.3)	1.000	
Smoking 5	0.2 (0.4)	0.2 (0.4)	0.2 (0.4)	0.1 (0.4)	1.000	

Significant differences are marked in bold. Abbreviations: C, control; f, female; m, male; HC, hydrocortisone;BMI, body mass index.

1 Level of education: 1–3 [1: basic, 2: high school, 3: college].

2 General wellbeing according to a 10-point visual analogue scale.

3 Alcohol consumption (number of times alcohol is consumed per week).

4 Drug consumption (YES/NO).

5 Smoking behaviour (YES/NO).

6 Mann-Whitney *U* test, exact statistic.

### 2.2 Imaging: Magnetic resonance image (MRI) data acquisition

All data were acquired with a 3T MR scanner (Discovery MR750, General Electric, Milwaukee, WI, USA) using an 8-channel head coil. This study is based on the rs-fMRI, included in a set of structural and functional acquisitions, following a specific protocol described previously (Van't Westeinde et al., 2019). Images were acquired with a planar echo imaging sequence (TR 2000 ms; TA echo time 30 ms; voxel size 3.0 × 3.0 × 3.0 mm^3^; 41 slices; thickness; 3.0 mm; flip angle; 70°). The acquisition time for the resting-state functional magnetic resonance images was 8 min (min) (70 min for the entire protocol). During the resting-state scan, participants were instructed to keep their eyes closed for the whole sequence.

### 2.3 Non-imaging statistics

#### 2.3.1 Demographic and clinical characteristics, assessment of executive functions and association between disease severity (phenotype) and visuo-spatial working memory

Participants also completed a battery of neuropsychological tests described elsewhere (Karlsson et al., 2017). The present study focused on executive functions, in particular on tests of visuo-spatial working memory (Span Board Forward and Backward) from the Wechsler Memory Scale (Wms-Iii, 2008); verbal working memory (Digit Span) from WAIS-IV (Wais-Iv, 2008) and inhibition/selective attention as assessed by the Stroop task (Golden and Freshwater, 1998). The variables we studied were tested for normality and homogeneity before each analysis. Analyses were performed using SPSS version 23 (IBM, Armonk, NY, USA). For the demographic and clinical data, one-way ANOVAs were conducted to compare CAH and controls on age, height, weight, BMI, education and self-reported mental wellbeing (using a 10-point visual analogue scale) at the time of scanning. A *p*-value threshold of 0.05 was used for significance determination. A non-parametric test (Mann-Whitney) was performed to compare CAH and controls for drug and alcohol use and smoking. ANOVAs were conducted to analyze between group differences in performance on tests assessing cognitive functions. Further, based on the observed group differences, we also tested the association between phenotype and visuo-spatial working memory performance (Span Board Forward) in the whole cohort and the CAH group separately, testing the linear relationships.

### 2.4 Resting-state functional MRI data analysis

#### 2.4.1 Data pre-processing

Resting-state functional magnetic resonance images were pre-processed using FMRIB́s Software Libraries version 5.0.11 (FMRIB Laboratory, University of Oxford, England, UK) (Smith et al., 2004). Briefly, the pre-processing included co-registration of each participant́s structural image with the functional image and head motion correction using MCFLIRT (Jenkinson et al., 2002), slice timing correction and brain extraction using a brain extraction tool (BET) (Smith, 2002), and finally, spatial smoothing with a Gaussian kernel of 5 mm full width.

Each participant's functional images were registered to the participant's structural images, using the FMRIBs Linear Image Registration Tool (FLIRT) (Jenkinson et al., 2002, Jenkinson and Smith, 2001) and to the standard space (MNI152) images using non-linear registration with a warp resolution of 10 mm.

After data pre-processing, the “aggressive” option of the ICA-based automatic removal of motion artifacts (ICA-AROMA) was used to identify and remove motion artifacts from the time series (Pruim et al., 2015, Pruim et al., 2015). ICA-AROMA is a robust strategy not requiring a study-specific training dataset. Motion-related components are automatically detected and removed from the initial data set through an ordinary least squares regression (Pruim et al., 2015).

#### 2.4.2 Independent component analysis

The cleaned individual data of resting-state was then fed into the spatial ICA to extract resting-state networks. ICA was performed using MELODIC v 3.15 software (FSL, Oxford, UK) (Beckmann et al., 2009).

First, we performed ICA separately for each of the two group conditions (patients with CAH and controls) to map the RSNs. The number of independent components was set to 60.

We then classified the resting-state networks based on their spatial similarity to the functional networks described in healthy people (Smith et al., 2009) by visually inspecting the aggregate spatial maps, time courses and the power spectrum. Next, the entire group of the pre-processed data, consisting of 69 participants, was concatenated and entered into an ICA group to identify common functional connectivity patterns for the whole cohort.

### 2.5 Dual regression analysis

Dual regression was used to regress the obtained group ICA components back into the individual participant’s space for all 69 participants (Beckmann et al., 2009). Next, group comparisons were performed using the FSL randomise tool (Winkler et al., 2014) with 5000 permutations to identify differences in resting-state connectivity between CAH and controls after controlling for age, sex and average frame displacement. The mean frame displacement for each subject has been obtained using FSL motion outliers tool (FMRIB) (Dipasquale et al., 2017). Significant clusters were identified with threshold-free cluster enhancement (TFCE) with a significance threshold of p < 0.05 (Winkler et al., 2014).

### 2.6 Association between functional connectivity and executive functions

We were further interested in testing the association between functional connectivity and executive functions (verbal- and visuo-spatial WM, inhibition) in patients with CAH in the whole brain. Impaired executive functions have been reported in adolescents and adult patients with CAH (Karlsson et al., 2017). Specifically, in our cohort we have observed reduced verbal and visuo-spatial working memory, as well as inhibition (Karlsson et al., 2017). To investigate the association between individual connectivity estimates from the ICA-based analyses with executive functions scores the FSL’s randomise tool was used (Winkler et al., 2014) in patients with CAH while controlling for age and sex. The inputs for the within-network analyses were the participant-specific time-series from the dual regression analysis.

### 2.7 Association between functional connectivity in the precuneus and CYP21A2 phenotype and medication dose

For the region in which a significant group difference in functional connectivity was found, we performed additional exploratory analyses to assess the relationship between functional connectivity of that region and medication dose (hydrocortisone equivalence in mg/m^2^ body surface at the time of scanning) in the CAH patient group and with disease severity as to phenotype in the entire cohort. Healthy controls were categorised as healthy phenotype.

First, using the cluster tool to define our cluster index and fslmaths to extract our mask (Smith et al., 2004) (FMRIB́s Software Libraries version 5.0.11) a mask was created for our ROI based on the significant outcome from the randomised analyses. Next, the mean time-series for each participant was extracted from this ROI using fslmeants (Smith et al., 2004), so that an estimate of mean functional connectivity of the precuneus was obtained per participant. Linear regression was then performed to test the association between functional connectivity and medication dose in the CAH patient group and between functional connectivity and phenotype in the entire cohort.

The phenotype was grouped based on severity: SW, SV and non-classic (NC) CAH. Patients with NC CAH were excluded from the association analysis due to small sample size (n = 2). The control group was also included as a “healthy” phenotype.

### 2.8 Association between functional connectivity of the precuneus and visuo-spatial working memory

In addition, we tested the relationship between functional connectivity in our ROI and performance on a test assessing visual-spatial working memory in patients and controls separately, testing the linear relationships.

### 2.9 Demographic and clinical characteristics, assessment of executive functions and association between disease severity (phenotype) and visuo-spatial working memory

Patients with CAH were older (approximately 3 years) than controls (p = 0.001) (Table 1). Moreover, as a group they were shorter (p = 0.002) and had a slightly higher body mass index (BMI) (p = 0.044) than the control group.

For the neuropsychological assessment, patients with CAH performed significantly worse than controls on the Span-board forward test (assessing visuo-spatial WM) (p = 0.001). Males with CAH performed worse than control males on the Stroop Interference Test (p = 0.044). We did not identify any other differences between groups (Table 2).

**Table 2:** **Average scores on cognitive measures assessing executive functions in patients with CAH and controls**. CAH patients performed worse on a test measuring visuo-spatial working memory (WMS-III Span Board Forward). Males with CAH performed worse on the Stroop Interference Test evaluating the ability to inhibit an over-learned response.

	*CAH* (n = 31)*	*C* *(n = 38)*	*[CAH vs C]*		*[CAH vs C]* *females*		*[CAH vs C]* *males*	
	*Mean (SD)*	*Mean (SD)*	F statistics	*p*	F statistics	*p*	F statistics	*p*
Executive functions								
WAIS‐IV - Digit Span (S)	9.5 (2.5)	10.5 (2.4)	1.64	0.15	0.65	0.362	1.04	0.245
WMS-III - Span Board Forward (T)	9.2 (2.1)	11.0 (2.6)	5.75	**0.001**	2.72	0.052	4.26	**0.008**
WMS-III - Span Board Backward (T)	11.5 (2.1)	12.4 (1.7)	1.42	0.205	1.47	0.148	0.76	0.772
Stroop interference (T)	52.6 (10.1)	54.3 (11.1)	1.83	0.101	1.28	0.770	4.55	**0.044**

Significant differences are marked in bold.

*One CAH participant did not complete the cognitive tests.

WAIS, Wechsler Adult Intelligence Scale; WMS, Wechsler Memory Scale.

S – scaled score; T- T score.

**Fig. 1:**
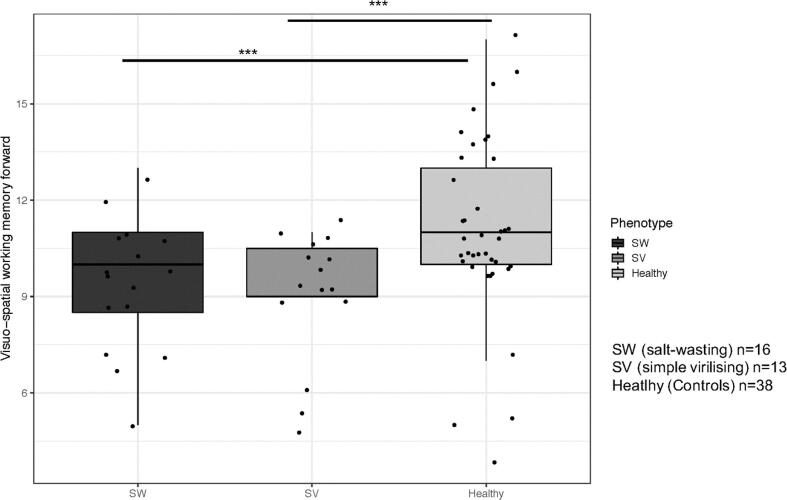
Association between CAH phenotype and visuo-spatial working memory performance (Span Board forward).

A linear association was confirmed in the whole cohort between phenotype and visuo-spatial working memory performance (Span board, forward (B = −1.152; *p* = 0.009)). Post-hoc analyses showed that there were no differences between SW and SV CAH groups. Both phenotype groups (SV CAH (B = −1.94, p < 0.001) and SW CAH (B = −2.27, p < 0.001)) performed worse on the visuo-spatial working memory test compared to controls (Fig. 1).

The figure illustrates the association between phenotype and visuo-spatial working memory performance. Both phenotype groups SV CAH and SW CAH performed worse on the visuo-spatial working memory test compared to controls. Each dot represents an individual score. The whiskers show the largest values within 1.5 times the interquartile ranges, above the third quartile (75%, the upper limit of the box) for the upward whisker and below the first quartile (25%, the lower limit of the box) for the downward whisker. The line in the middle represents the median.

### 2.10 Group-independent component analysis

**Fig. 2:**
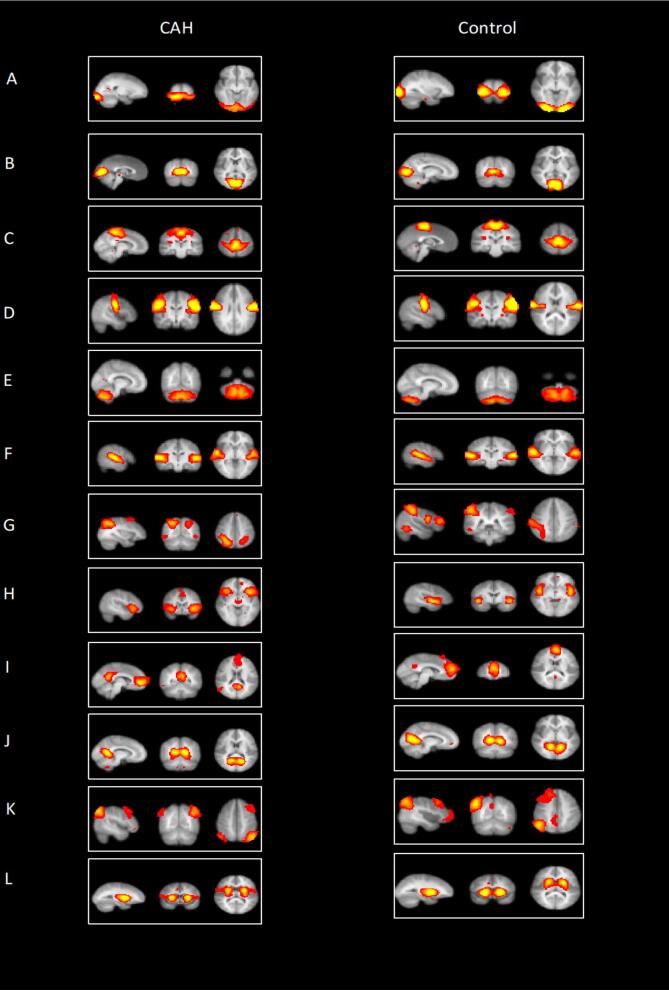
Resting-state functional networks.

Twenty-three of 60 components for the patients with CAH and 29 of 60 for the controls were considered to reflect the RSNs previously described in the literature for healthy individuals (Smith et al., 2009, Damoiseaux et al., 2006). All other plausible components were judged to be noise-related artifacts, such as ventricles, cerebral spinal fluid, head motion, blood vessels or components that did not match any networks. We identified 12 networks based on their spatial configuration (Smith et al., 2009, Damoiseaux et al., 2006). The obtained RSNs included the visual (pole and medial), motor leg-hand and motor-face regions, cerebellum, auditory/language, dorsal attention, salience, default mode (medial PFC and PCC), executive and basal-ganglia networks (Fig. 2).

Twelve functional networks were identified in the cohort during rest: (A) Visual (pole), (B) visual (medial), (C) motor leg-hand, (D) motor face regions (E) cerebellum, (F) auditory/language, (G) dorsal attention, (H) salience network, (I) default mode network (mPFC, medial prefrontal cortex), (J) default mode network (posterior cingulate cortex), (K) executive network, (L) (basal-ganglia).

### 2.11 Dual regression analysis

**Fig. 3:**
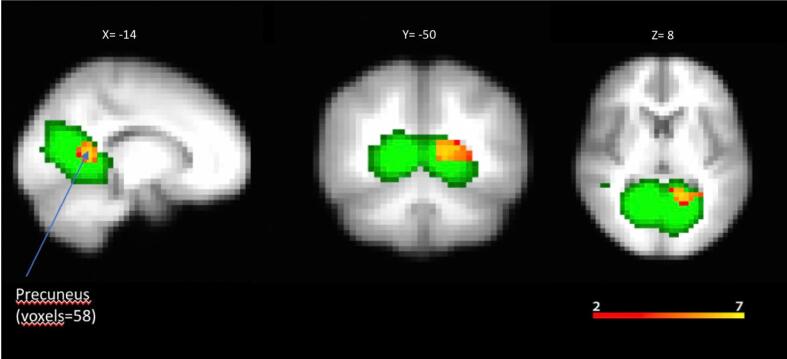
Within network connectivity: independent component analysis. Functional network with increased functional connectivity in patients with CAH.

The CAH group showed a pattern of increased connectivity in the left precuneus cortex compared with the control group (voxels = 53; x = −50; y = −14; z = 8; p = 0.008) (Fig. 3).

The DMN, extract using group ICA, is overlaid on the average MNI-152 brain template in green colour.

Within DMN, significant group differences were observed for a cluster encompassing the precuneus region (peak MNI coordinates: X = −14, Y = −50, Z = 8; P = 0.008).

### 2.12 Association between resting-state functional connectivity and executive functions

No association was identified between whole-brain functional connectivity and executive functions (verbal and visuospatial WM, inhibition) in patients with CAH.

### 2.13 Association between resting-state functional connectivity of the precuneus and CYP21A2 phenotype and medication dose

**Fig. 4:**
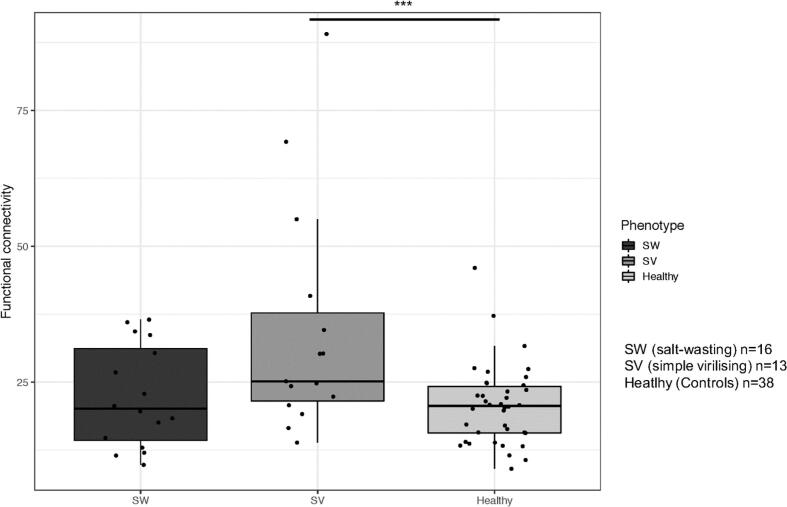
Association between functional connectivity of the precuneus and CAH phenotype.

We observed a linear association between phenotype and functional connectivity (B = 5.65, p = 0.009). Post-hoc analyses showed that there were no differences between patients with SW and SV CAH. However, only individuals with SV CAH had significantly stronger connectivity compared to controls (B = 17.65, p < 0.001), while individuals with SW CAH did not differ from control subjects (B = 4.91, p = 0.10) (Fig. 4).

Total hydrocortisone replacement dose did not correlate with functional connectivity in the precuneus.

The figure illustrates the association between functional connectivity of the precuneus and phenotype for the whole cohort (CAH and Control group). We observed a significant linear association between phenotype and functional connectivity. Only patients with SV CAH had higher functional connectivity compared to controls. The whiskers show the largest values within 1.5 times the interquartile ranges, above the third quartile (75%, the upper limit of the box) for the upward whisker and below the first quartile (25%, the lower limit of the box) for the downward whisker. The line in the middle represents the median.

### 2.14 Association between functional connectivity of the precuneus and visuo-spatial working memory

There was no significant linear relationship between functional connectivity in the precuneus and performance on the forward visuo-spatial working memory task in the CAH or whole cohort.

## 3 Discussion

For the first time, differences in resting-state functional connectivity in participants with CAH compared with healthy controls have been examined. Using group ICA and dual regression analysis, increased functional connectivity was found in patients with CAH in the left precuneus cortex. In addition, patients with CAH performed worse on a test assessing visuo-spatial working memory (Span Board Forward) and males with CAH performed worse on the Stroop Interference Test. However, the functional connectivity in the whole-brain resting-state networks did not correlate with executive function outcomes in patients with CAH. Looking at the ROI (precuneus), we did not identify any link between functional connectivity in the precuneus and performance on the visuo-spatial working memory task in the CAH patients. However, we observed a linear association between phenotype and functional connectivity. Post-hoc tests within the ROI showed that only patients with SV CAH had stronger connectivity compared to controls. Further, while both SW and SV CAH performed worse on a working memory task (Span Board forward) compared to controls, functional connectivity in the precuneus was not associated with any executive function performance. Total hydrocortisone replacement dose at the time of testing did not correlate with functional connectivity in the precuneus.

The DMN structurally consists of the precuneus, posterior cingulate cortex and medial prefrontal cortex. In healthy people this network shows a decreased connectivity during attention-demanding tasks (McKiernan et al., 2003) and increased connectivity during rest (Fox and Raichle, 2007) when not engaged in the task but in self-referential thinking. The precuneus, located in the posteromedial part of the parietal lobe, has been defined as the core node or hub of the DMN (Fransson and Marrelec, 2008). It is characterised by high energetic demand, consuming 35% more glucose than the other hubs (Gusnard et al., 2001). Indeed, the precuneus is functionally connected with the frontal cortex and is part of the central executive network (Eriksson et al., 2015). During working memory performance, it is involved in shifting attention between different locations; during rest, it is engaged in such processes as self-referential thinking or consolidation of episodic memory (Cavanna and Trimble, 2006). Thus, the precuneus is involved in two of the three networks of the triple network core (Menon, 2011). These networks include the salience network, central executive and DMN. Aberrant connectivity in one of the networks might affect the engagement of the other networks, leading to affect and cognitive functioning problems. However, no correlation was observed between precuneus connectivity at rest and performance on the cognitive tasks in our study. Thus, even though patients with CAH performed worse on a test assessing visuo-spatial working memory (WMS-III Span Board Forward) and males with CAH performed worse on the Stroop Interference Test, this poor performance did not appear to be related to the activity of the precuneus. The apparent lack of association could be attributable to the small sample size or suggests that higher activity in the precuneus at rest does not lead to problems with working memory in patients with CAH. Moreover, we recently found increased activity in a more dorsal part of the precuneus in males with CAH during working memory performance (Van't Westeinde, submitted). This could reflect that increased activity in the precuneus is needed in patients to maintain equal task performance. Potentially, increased activity of the more ventral region of the precuneus at rest could be part of this compensation mechanism.

Interestingly, we found a linear association between functional connectivity and phenotype. Patients with the SV phenotype showed higher functional connectivity in the precuneus. The current cohort is too small to do meaningful statistical tests to assess whether these functional connectivity changes per genotype group are relevant for working memory performance. However, both SW and SV CAH performed worse on a working memory task (Span Board forward) compared to controls.

All our subjects were diagnosed through the neonatal screening program, but it is possible that episodes of hyponatremia or hypoglycemia later in life may have affected cognition. Studies on larger groups are needed to investigate these relationships further.

A relationship is claimed to exist between the structure of the brain and functional connectivity (van den Heuvel et al., 2009). Thus, the observed higher connectivity in the precuneus might be linked to underlying changes in structure in the involved regions. Our recent study on brain structure in patients with CAH (Van't Westeinde et al., 2019) reported alterations in grey and white matter structure. Specifically, we found reduced volume in the precuneus, as well as reduced cortical thickness in the middle frontal gyrus and the parietal and superior occipital cortex. A higher activity at rest in the precuneus in CAH patients might be interpreted as a compensation mechanism for reduced volume.

One explanation for the sensitivity of the precuneus to functional and structural alterations in CAH is that this major brain hub might be more vulnerable to hormonal imbalances because of its high metabolic rate (Cavanna and Trimble, 2006). In this connection, alterations in some RSNs have been reported in patients with Cushing’s disease in remission, a condition characterized by cortisol excess (Stomby et al., 2019, Jiang et al., 2017). More specifically, in comparison with the healthy controls, Cushing’s disease patients showed elevated functional connectivity within the DMN. Notably, a higher cortisol level in patients with Cushing’s disease correlates with a lower metabolic rate in the precuneus and middle frontal gyrus (Lin et al., 2017).

Increased functional activity in the DMN has also been detected in resting-state studies of patients with stress-related disorders (Zhu et al., 2015). These disorders are characterized by dysregulated HPA axis functions, which might lead to hypercortisolism. Cortisol imbalances through altered glucose metabolism may affect functional neuronal activity. In our cohort, 83% of the patients had an adequate metabolic control, but we cannot exclude that the remaining 17% had a poor metabolic control.

## 4 Conclusion

Altered functional connectivity during rest in patients with CAH might reflect a functional reorganization in response to the CAH disease. In addition, the observed changes in functional connectivity might be dependent on phenotype. Treatment optimization might help patients to maintain optimal brain functioning. Certainly, more studies are needed to evaluate longitudinal changes in patients with CAH.

## 5 Limitations

Our study has some potential limitations. First, head movement may affect the connectivity estimates. To remove the motion-related signal from our data we used a strict procedure. After the data pre-processing, in addition to the standard motion correction with volume realignment, the “aggressive” option of ICA-AROMA was used to identify and remove motion artifacts from the time series as well explained in the Methods section under “Resting-state functional MRI data analysis.”

A second potential limitation is that our cohort includes both women and men with CAH and patients with different genotypes, but the group size limits a more detailed evaluation of sex- and genotype-specific differences in functional connectivity. We included age as a covariate in the statistical analysis. However, because the CAH group was a few years older than the control group, this control may underestimate the effects observed in the CAH group. Larger study groups with wider age ranges would enable investigations of the impact of different genotypes/phenotypes in CAH, accumulated GC load and multiple adrenal crises on resting-state functional connectivity.

## Funding

This work was supported by the Marianne and Marcus Wallenberg Foundation, the International Fund raising for Congenital Adrenal Hyperplasia (IFCAH)/European Society for Pediatric Endocrinology (ESPE), the Stockholm County Council (ALF-SLL), Swedish Research Council (DNR 2021-02440), Region Stockholm (clinical research appointment DNR RS 2019-1140 to S.L.), the Foundations of Lisa and Johan Grönberg, Stiftelsen Frimurare Barnhuset i Stockholm, Samariten, Jerringfonden, Sällskapet Barnavård, Wera Ekströms stiftelse för Pediatrikforskning and the Foundation for Research and Education in Pediatric Endocrinology.

## CRediT authorship contribution statement

**Valeria Messina:** Conceptualization, Formal analysis, Writing – original draft. **Annelies van't Westeinde:** Formal analysis, Writing – review & editing. **Nelly Padilla:** Supervision, Conceptualization, Writing – review & editing. **Svetlana Lajic:** Supervision, Writing – review & editing.

## Declaration of Competing Interest

The authors declare that they have no known competing financial interests or personal relationships that could have appeared to influence the work reported in this paper.
